# Intestinal organoids for assessing nutrient transport, sensing and incretin secretion

**DOI:** 10.1038/srep16831

**Published:** 2015-11-19

**Authors:** Tamara Zietek, Eva Rath, Dirk Haller, Hannelore Daniel

**Affiliations:** 1 Department of Nutritional Physiology, Technische Universität München, 85350 Freising, Germany; 2 ZIEL—Institute for Food & Health, 85350 Freising, Germany; 3 Chair of Nutrition and Immunology, Technische Universität München, 85350 Freising, Germany

## Abstract

Intestinal nutrient transport and sensing are of emerging interest in research on obesity and diabetes and as drug targets. Appropriate *in vitro* models are lacking that allow both, studies on transport processes as well as sensing and subsequent incretin hormone secretion including intracellular signaling. We here demonstrate that murine small-intestinal organoids are the first *in vitro* model system enabling concurrent investigations of nutrient and drug transport, sensing and incretin hormone secretion as well as fluorescent live-cell imaging of intracellular signaling processes. By generating organoid cultures from wild type mice and animals lacking different nutrient transporters, we show that organoids preserve the main phenotypic features and functional characteristics of the intestine. This turns them into the best *in vitro* model currently available and opens new avenues for basic as well as medical research.

There is growing interest in intestinal nutrient transport, sensing and hormone secretion because of their central roles in metabolic disorders such as obesity and type 2 diabetes^1^. Nutrient transporters and receptors have been identified as sensors for glucagon-like peptide 1 (GLP-1) secretion, and the impressive antidiabetic effects of bariatric surgery are largely attributed to altered nutrient sensing processes in small intestine (SI)^2^,^3^. Moreover, mimetics of GLP-1 are successfully used in diabetes therapy^4^,^5^. Currently, intestinal epithelial cell (IEC) lines and enteroendocrine cell (EEC) lines are the prime *in vitro* model systems to characterize intestinal transport processes for nutrients and drugs or to assess secretion of gut hormones in response to luminal stimuli. Their ease in handling is counterbalanced by special phenotypic features since the cells are almost all tumor-derived. These cultures of course cannot simulate the complexity of the intestinal epithelium with multiple cell types and region-specific architecture and hormone expression patterns of all subtypes of EEC^6^,^7^. Primary IEC culture^8^ seems therefore a better approach to study intestinal functions *in vitro* but they usually have only poorly differentiated enterocytes not suitable for assessing transport functions. Moreover, primary IEC are limited by short-term culture and their generation requires rather large number of animals.

In 2009, Sato *et al.* reported the generation and long-term *in vitro* cultivation of intestinal organoids (also referred to as “enteroids“^9^) of human and murine origin^10^,^11^. Containing all cell types of the intestinal epithelium including stem cells as well as EEC^12^, organoids resemble the main characteristics of the mammalian intestine and offer many applications for research, not only for regenerative medicine and transplantation^13^,^14^ but also for basic research^15^.

We explored whether murine SI organoids are applicable as an *in vitro* model for nutrient transport and sensing studies. Moreover, incretin hormone secretion was tested in response to different luminal stimuli combined with fluorescent live-cell imaging of intracellular signaling.

## Results

### Transport of nutrients and drugs in intestinal organoids

**Figure 1:**
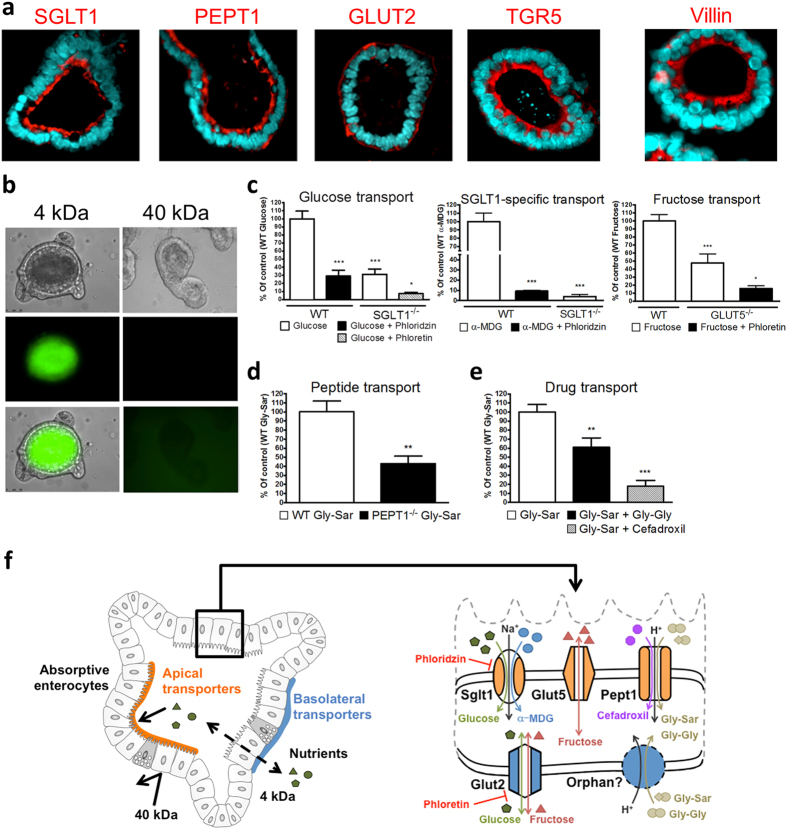
Presence and functionality of nutrient transporters in SI organoids. **(a)** Immunofluorescent stainings of nutrient transporters (red), the bile acid receptor TGR5 (red) and villin (red), nuclei (blue). **(b)** Diffusion pattern of FITC-Dextran (4 kDa) and FITC-Dextran (40 kDa). Bottom right: increased light exposure showing lack of diffusion. **(c)** Transport of radiolabeled glucose, α-MDG and fructose in organoids derived from WT, SGLT1^−/−^ or GLUT5^−/−^ mice. Phloridzin: SGLT1-inhibitor, Phloretin: GLUT-inhibitor **(d)** Transport of the radiolabeled dipeptide Gly-Sar in organoids from WT or PEPT1^−/−^ mice. **(e)** Competitive inhibition of radiolabeled Gly-Sar (5 mM) uptake by the dipeptide Gly-Gly (10 mM) and the β-lactam-antibiotic cefadroxil (20 mM). **(f)** Overview of the transport processes.

We first determined the expression and localization of some known solute carriers as well as of the bile acid receptor TGR5 in polarized SI organoids by immunofluorescent staining (Fig. 1a and Supplementary Fig. S1). Crossly matching the *in vivo* situation, the sodium-dependent glucose transporter SGLT1/SLC5A1, the proton-coupled peptide transporter PEPT1/SLC15A1 and TGR5 were detected with brush border localization whereas the facilitated glucose transporter GLUT2 was found on the basolateral side of the epithelium (Fig. 1a). To assess the specificity of the transport process, we employed organoids prepared from mice lacking either SGLT1 or PEPT1. Transport function was determined with radiolabeled substrates and quantification of tracer uptake into organoid enterocytes. Since the (inner) luminal side of the intestinal organoid is not directly accessible *in vitro*, we investigated the permeability character of the epithelial layer by use of fluorescent tracers. Sodium-fluorescein and FITC-Dextran (FD4) quickly labeled the organoid lumen demonstrating a high permeability for compounds of up to 4 kDa such as glucose, peptides and fatty acids. In contrast, FITC-Dextran with a size of 40 kDa failed to enter the organoid lumen (Fig. 1b and Supplementary Fig. S1).

Functional characterization of monosaccharide transport in SI organoids was performed with radiolabeled D-glucose, 1-O-methyl-alpha-D-glucopyranoside (α-MDG) and D-fructose (Fig. 1c). Glucose serves as a substrate for both, apical and basolateral GLUT transporters, except for GLUT5 which is specific for D-fructose^16^. In contrast to glucose, α-MDG is a specific, non-metabolizable substrate of the rheogenic SGLT1 transporter exclusively found in apical membranes^17^. Uptake of D-glucose and α-MDG transport were blunted by use of the SGLT1 inhibitor phloridzin and, to a similar extent, in organoids derived from SGLT1-deficient mice. The remaining glucose transport in SGLT1^−/−^ organoids was inhibited almost completely by the chalcone phloretin, and thus is attributable to GLUT proteins with GLUT2 found in the basolateral membrane of enterocytes providing glucose exit into circulation. However, as a uniporter, GLUT2 can also import glucose into tissues across the basolateral side when a gradient exists. GLUT2 also mediates facilitated diffusion of D-fructose in enterocytes, while fructose from the luminal side is taken up via GLUT5^16^. Accordingly, we observed uptake of fructose to be reduced by half in organoids generated from mice lacking GLUT5 compared to those from wildtype (WT) controls with phloretin diminishing the residual fructose uptake in GLUT5^−/−^ organoids strongly (Fig. 1c).

In assessing peptide absorption, organoids were exposed to the radiolabeled dipeptide glycyl-sarcosin (Gly-Sar), a hydrolysis-resistant model substrate of PEPT1. In addition to di- and tripeptides, PEPT1 also facilitates the oral absorption of numerous peptidomimetic drugs including some antibiotics and ACE inhibitors^18^. Although not identified on a genetic basis, a system for basolateral peptide uptake with similar features as PEPT1 has been demonstrated^19^. Gly-Sar uptake into organoids derived from PEPT1-deficient mice was reduced by around 60% suggesting the remainder to occur via basolateral influx. Moreover, Gly-Sar uptake in WT organoids was competitively inhibited by the dipeptide glycyl-glycine (Gly-Gly) as well as by the aminocephalosporin antibiotic cefadroxil (Fig. 1d,e). Taken together, these findings demonstrate that organoids prepared from the small intestine of mice represent a suitable *in vitro* model for studying intestinal transport processes for nutrients and drugs (Fig. 1f).

### Nutrient sensing and incretin hormone secretion in intestinal organoids

In recent years, intestinal transporters such as SGLT1 and PEPT1 were shown to have a role as nutrient sensors and mediators of gut hormone secretion from EEC. Amongst all gut hormones, the incretins GLP-1 and glucose-dependent insulinotropic polypeptide (GIP) are of particular interest due to their effect on insulin secretion and ß-cell protection. In addition to the glucose and peptide sensors SGLT1 and PEPT1^20^,^21^, the bile acid deoxycholic acid (DCA) acting via TGR5 was shown to effectively stimulate GLP-1 secretion^22^,^23^. These intestinal sensing pathways have been identified as promising target for the treatment of type 2 diabetes and synthetic agonists were shown to increase incretin secretion and improve blood glucose control^24^. It has recently been demonstrated that incretin hormone release can be measured from intestinal organoid cultures and that L-cell number and consequently incretin secretion increases upon treatment of organoids with short-chain fatty acids or with the Notch inhibitor DBZ^12^,^25^. In order to demonstrate that organoid cultures can be used as *in vitro* model for gut sensing research, we assessed if significant incretin responses to different stimuli (compared to basal incretin secretion) can be measured. We furthermore specified nutrient-induced incretin release and the responsible sensor proteins by use of organoids derived from mice lacking the transporters that serve as nutrient sensors.

**Figure 2:**
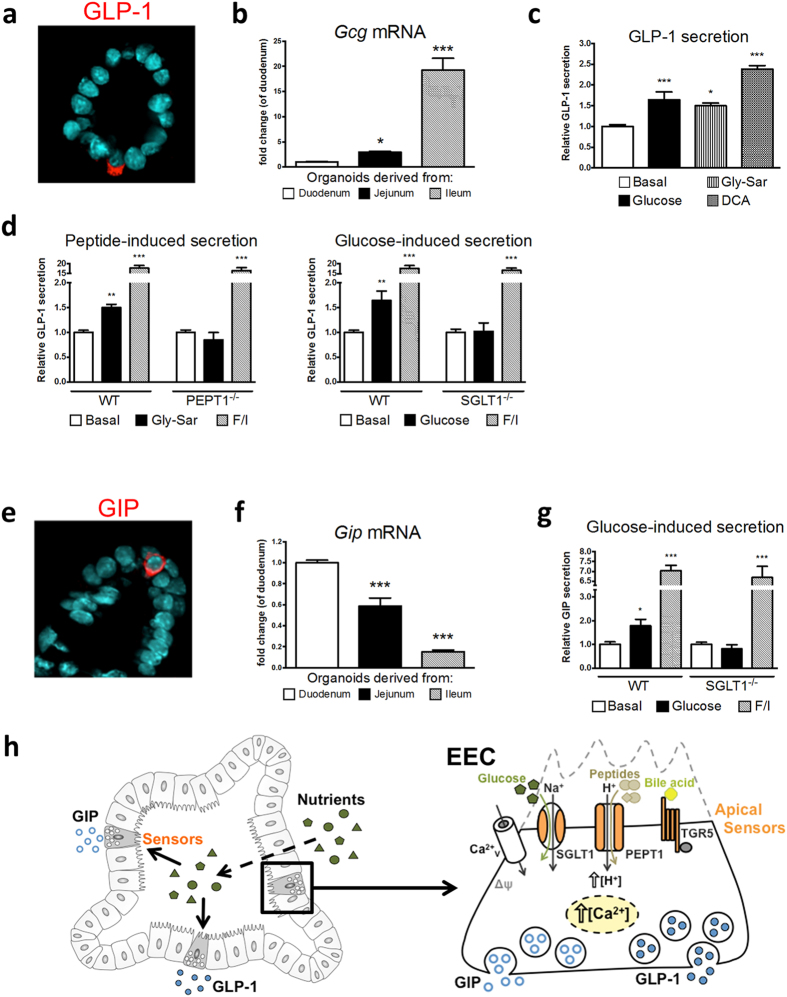
Nutrient sensing and incretin secretion in SI organoids. **(a)** Immunofluorescent staining of a GLP-1-containing enteroendocrine cell (red), nuclei (blue). **(b)** *Gcg* (encoding GLP-1) mRNA expression in organoids derived from different parts of the SI. Organoids stated as derived from duodenum contained a part of the proximal jejunum. **(c)** Relative GLP-1 secretion from SI organoids in response to different physiological stimuli. **(d)** Relative GLP-1 secretion in response to the dipeptide Gly-Sar in WT or PEPT1^−/−^ organoids, and to glucose in WT or SGLT1^−/−^ organoids. **(e)** Immunofluorescent staining of a GIP-containing enteroendocrine cell (red), nuclei (blue). **(f)** *Gip* mRNA expression in organoids derived from different parts of the SI. **(g)** Glucose-stimulated GIP release from WT or SGLT1^−/−^ organoids. **(h)** Overview of incretin secretion.

Immunofluorescent staining confirmed the presence of GLP-1- and GIP-containing EEC in SI organoids (Fig. 2a,e). It is established that the number of GLP-1-containing cells increases from proximal SI/duodenum to distal SI/ileum and *vice versa* for GIP-containing cells. When preparing organoids from different parts of the SI, we obtained the same expression pattern for the *Gcg* (encoding GLP-1) and *Gip* mRNA showing that physiology is preserved *in vitro* (Fig. 2b,f). A region-specific expression pattern of intestinal specific genes in organoids derived from different parts of the intestine including some transporters was recently reported^26^. We, as others, have used organoids from duodenum/proximal SI (Supplementary video 1) as they display the highest culture efficiency. We show that these organoids are useful for assessing GLP-1 responses to glucose, dipeptides and DCA (Fig. 2c). In line with our previous results^17^,^20^, GLP-1 responses to glucose and Gly-Sar were blunted in organoids prepared from SGLT1^−/−^ and PEPT1^−/−^ animals, respectively (Fig. 2d). Although sweet-taste receptors which are discussed to contribute to glucose-induced incretin secretion^27^ might be expressed by organoids, the lack of incretin release in absence of SGLT1 matches previous reports including animal studies^17^,^21^,^28^. Glucose is a potent secretagogue for GLP-1 and also for GIP^21^. Accordingly, we observed a robust GIP response upon glucose stimulation in WT, but not in SGLT1^−/−^ organoids (Fig. 2g), demonstrating that SI organoids are a useful tool for studies on both incretin hormones (Fig. 2h). In all experiments, a mixture of forskolin/3-isobutyl-1-methylxanthine served as a control for maximal hormone output.

### Fluorescent live-cell imaging of calcium fluxes and intracellular acidification

**Figure 3:**
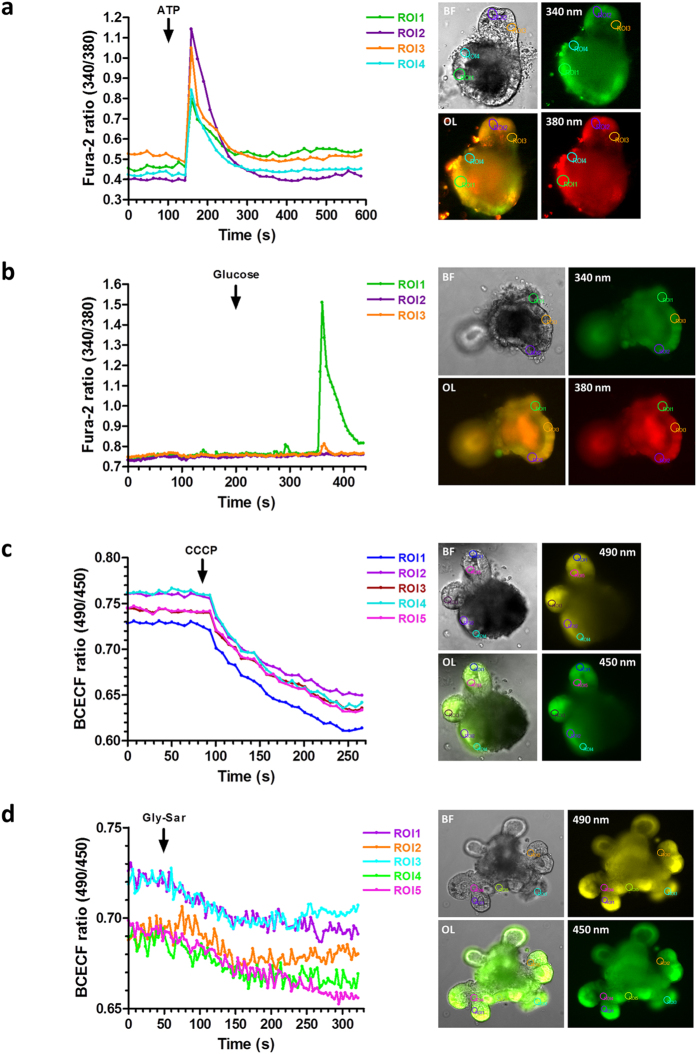
Live-cell imaging of intracellular calcium and acidification in SI organoids using the fluorescent indicators Fura-2-AM and BCECF-AM. (**a**) Left: uniform calcium responses to ATP (100 μM), measured in 4 distinct ROI (regions of interest). Right: corresponding pictures of the organoid; 340/380 nm (excitation wavelengths), BF: bright-field, OL: overlay. (**b**) Calcium response to glucose (50 mM), restricted to one ROI (3 without response) and corresponding pictures. (**c**) Left: uniform increases in intracellular proton levels induced by the protonophore CCCP (40 μM). Right: corresponding pictures of the organoids; 490/450 nm (excitation wavelengths). (**d**) Intracellular acidification induced by the dipeptide Gly-Sar (50 mM).

**Figure 4:**
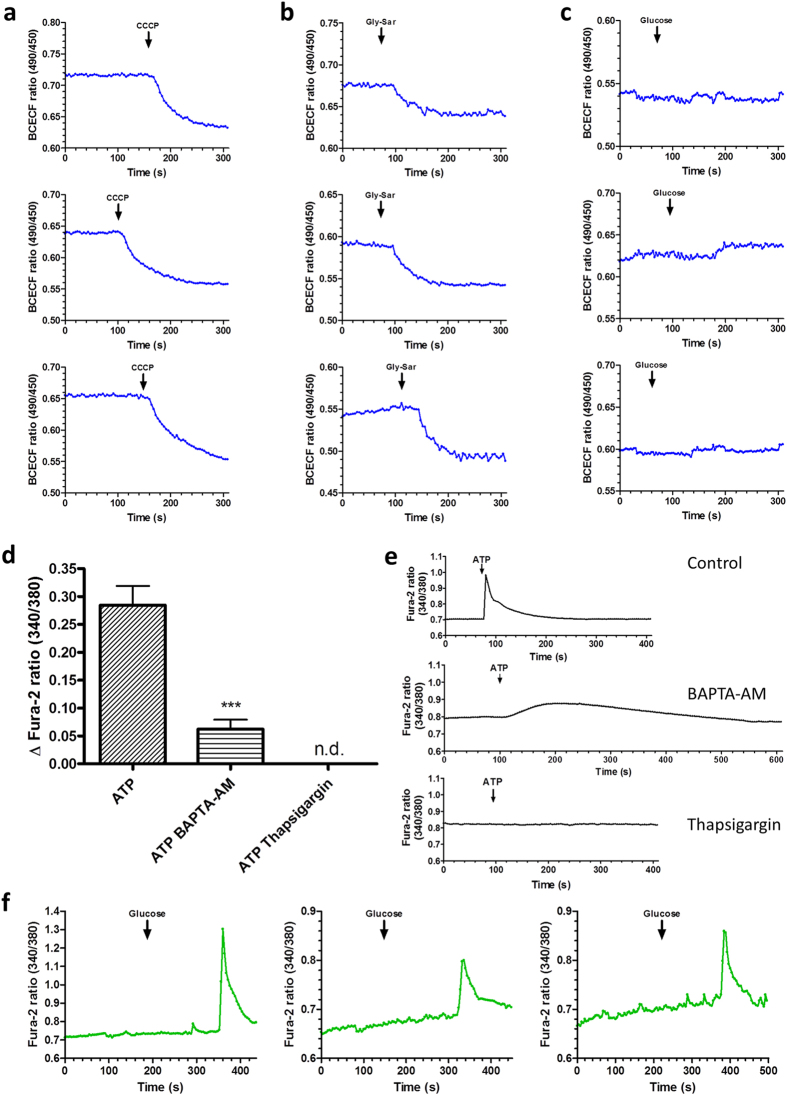
Reproducibility of imaging data demonstrated by measurements in different WT organoids, and inhibition of ATP-induced intracellular calcium increase. (**a**) CCCP (40 μM)-induced intracellular acidification in three representative organoids. (**b**) Intracellular acidification upon Gly-Sar (50 mM) treatment in three representative organoids. (**c**) No change in intracellular proton levels was observed upon addition of 50 mM glucose as a “negative control”, three representative organoids. (**d**) Inhibition of ATP-induced intracellular calcium increase by pretreatment with the cell-permeable calcium chelator BAPTA-AM (20 μM) or with the SERCA pump inhibitor thapsigargin (1 μM). Cultures were preincubated with BAPTA-AM or thapsigargin 45 min. prior to the calcium imaging experiments. N = 10–20 organoids per group, data are presented as mean ± SEM; statistical analysis: unpaired t-test with Welsh’s correction. (**e**) Representative time courses of ATP-induced calcium signals. (**f**) Intracellular calcium increase upon glucose (50 mM) treatment in three different organoids. Each measurement was performed with a different WT organoid (derived from independent cultures). For data analysis (**a**–**e**), the whole organoid was selected, and no background correction was applied. For analysis of glucose-induced ATP responses (**f**), the distinct ROI (region of interest) responding to glucose was selected, no background correction.

We employed live-cell imaging using fluorescent probes for visualization of changes in intracellular calcium levels and intracellular acidification. An elevation of the cytosolic calcium concentration in response to nutrient sensing is a common mediator in exocytosis of gut hormones. Moreover, transporter regulation such as for PEPT1^29^ and GLUT2^30^ was shown to depend on intracellular calcium levels. Calcium imaging is furthermore frequently used in pharmacological drug screening to detect activation of receptors by putative ligands in cell-based assay systems. SGLT1 and PEPT1 mediate cation influx in their transport cycles and thus cause a substrate-induced membrane depolarization which can cause voltage-gated L-type calcium channels to open and leads to hormone release from EEC^20^. Using the calcium-indicator Fura-2-AM and the pH-indicator BCECF-AM, we visualized changes in intracellular calcium levels and acidification, respectively, in epithelial cells of SI organoids. As expected based on literature, with both indicators, robust signals were obtained for ATP-dependent calcium changes^31^,^32^,^33^ and also for carbonyl cyanide *m*-chlorophenyl hydrazine (CCCP, an ionophore) mediated acidification. It was demonstrated in isolated murine villi that dipeptides lead to intracellular acidification in all enterocytes^34^, while glucose was shown to induce a calcium response in murine enteroendocrine L-cells but not in non-L-cells^8^. We thus demonstrate that physiological stimuli such as D-glucose and Gly-Sar cause the expected intracellular changes in the organoid cultures (Fig. 3, Supplementary Fig. S2 and Supplementary videos S2 and S3). Glucose-responsive cells were very rare in our cultures and distinct regions displaying a calcium increase upon glucose stimulation were observed in 3 out of 10 organoids (Fig. 4f). This is however not surprising given that glucose was reported to induce a calcium increase solely in enteroendocrine L-cells^8^ which are very rare in our organoid cultures as they also are in native (proximal) small intestine. As expected, glucose does not lead to an intracellular acidification of enterocytes (Fig. 4c). Moreover, intracellular calcium signaling could be specified by pretreatment of organoid cultures with the cell-permeable calcium chelator BAPTA-AM, and with the SERCA-inhibitor Thapsigargin blocking IP_3_-dependent release of calcium from intracellular stores. We found the ATP-induced rise in intracellular calcium to be markedly inhibited upon pretreatment with BAPTA-AM or Thapsigargin as it is described for purinergic receptors activated by ATP^35^,^36^ (Fig. 4d,e). In contrast to poor dye-loading frequently observed in cell cultures, SI organoids displayed an excellent loading efficiency for the fluorescent indicators (see Fig. 3).

## Conclusion

Taken together, we demonstrate that organoids prepared from small intestine of mice preserve in culture the main features and can be used for studying nutrient transport, sensing and hormone secretion. The major and unique advantage of the organoid system for nutrient sensing research (over other *in vitro* models) is the opportunity to investigate simultaneously GIP and GLP-1 expression, localization and activity of the sensor proteins and intracellular signaling processes that couple nutrient sensing to incretin release. There is no cell system available that allows the concurrent investigations of all these processes. Another prominent advantage over standard cell culture is the ease and fast generation of organoids from animals lacking certain (transporter) proteins as compared to constructing cell-lines with knockout or knock-down.

Primary intestinal monolayer cultures are currently the only model allowing concurrent analyses of GIP and GLP-1 secretion as well as calcium signaling events and, as the organoids, they can be easily generated from knockout mice as shown previously^20^,^28^. The primary cultures yet have two major disadvantages over organoid cultures: (i) they cannot be passaged which increases the number of laboratory animals needed and (ii) they cannot be cultivated longer than a few days, hence enterocytes are poorly differentiated. As an *in vitro* model, intestinal organoids come closest to physiology and provide thus a wide range of application areas from basic gastrointestinal research over nutrition to drug bioavailability studies and sensory science with imaging of intracellular signaling processes. Human enteroid monolayer cultures recently described might constitute another promising *in vitro* system for functional studies of gastrointestinal processes^37^. Even though animal studies cannot be replaced by SI organoid cultures in all cases, this novel model system has the potential to reduce the number of *in vivo* studies, as it gets closer to physiology than any other *in vitro* model before.

## Methods

### Mice

Mice used for small intestinal crypt isolation were 8–18 weeks of age. Wild type (WT) C57BL/6 mice, PEPT1^−/−^ mice (C57BL/6 background)^38^, SGLT1^−/−^ mice (C57BL/6 background)^21^ and GLUT5^−/−^ mice (C57BL/6 background)^39^ were bred and kept in the animal facilities of the Institute for Food & Health (ZIEL, Freising, Germany). Mice were sacrificed by CO_2_ and small intestine was removed. All animal procedures were approved by the Bavarian Animal Care and Use Committee and all experiments were performed in accordance with relevant guidelines and regulations.

### Crypt isolation

Small intestinal crypts were isolated as previously described^10^ with minimal modifications. Briefly, the small intestine was dissected, cut into half and the proximal part was inverted and washed with cold PBS (pH 7.4). For longitudinal studies, the small intestine was cut into three parts equal in size; the proximal part was considered as duodenum (albeit it contains a part of the proximal jejunum), the middle part as jejunum and the distal part as ileum, respectively. Tissue pieces were incubated with 2 mM EDTA in PBS for 30 min at 4 °C. After removal of EDTA, tissue pieces were shaken vigorously in PBS several times to obtain supernatant fractions enriched in crypts. Appropriate fractions were passed through a 70 μm cell strainer and centrifuged at 300 *g* for 5 min at 4 °C. Pelleted crypts were carefully resuspended in Matrigel.

### Organoid culture

Isolated crypts (Supplementary video S1) and organoids were cultured as previously described^10^ with minimal modifications. Briefly, 25 μl of Matrigel containing crypts/organoids were plated in the center of wells in a 48-well plate. After polymerization at 37 °C, 300 μl of crypt culture medium (CCM) containing growth factors (EGF, R-Spondin 1, Noggin) was added. CCM was changed twice a week. After 7 days, organoid cultures were passaged. CCM was removed and Matrigel including organoids was dissolved with cold PBS. Following centrifugation at 300 *g* for 5 min at 4 °C, supernatant was removed and pelleted organoids were carefully resuspended in fresh Matrigel. All experiments were performed after two passages.

### Paraffin embedding and immunohistochemical labeling

For paraffin embedding, organoids were harvested by dissolving Matrigel including organoids with cold PBS. Following centrifugation at 300 *g* for 5 min at 4 °C, supernatant was removed and pelleted organoids were carefully resuspended in pre-heated HistoGel (Thermo Scientific). 70 μl droplets were placed on pre-cooled glass slides and incubated at 4 °C for 5–10 min until turned solid. Subsequently, organoids were fixed in 4% neutral buffered formalin and embedded in paraffin. For immunofluorescent staining, paraffin-embedded organoids were cut into 5 μm sections using a Leica RM2255 and applied up onto polylysine coated slides, air dried at room temperature for 1 h and dried at 37 °C over night. Samples were deparaffinized and antigens were unmasked by 10 min incubation in boiling 10 mM sodium citrate buffer (pH 6.0) (Roth). Immunostaining was performed according to the protocol provided by Cell Signaling.

The following antibodies were used for immunofluorescence stainings: anti-SGLT1 (species rabbit, dilution 1:1000, custom-made by Pineda), anti-PEPT1 (species rabbit, dilution 1:1000, custom-made by Pineda), anti-GLUT2 (species goat, dilution 1:200, manufacturer: Santa Cruz), anti-TGR5 (species rabbit, dilution 1:200, manufacturer: Abcam), anti-Villin (species goat, dilution 1:1000, manufacturer: Santa Cruz), anti-E-Cadherin (species rabbit, dilution 1:1000, manufacturer: Santa Cruz), anti-GLP-1 (species goat, dilution 1:200, manufacturer: Santa Cruz), anti-GIP (species goat, dilution 1:200, manufacturer: Santa Cruz).

A Cy3 donkey anti-goat IgG (Abcam), dilution 1:500, a Cy3 donkey anti-rabbit IgG (Invitrogen), 1:250, an Alexa Fluor 488 donkey anti-goat IgG (Abcam), 1:500, and an Alexa Fluor 647 donkey anti-rabbit IgG (Abcam), 1:250, were used as secondary antibodies. Rabbit IgG and goat IgG (all Santa Cruz) were used as isotype controls. Specificity of custom-made PEPT1 and SGLT1 antibodies was verified in intestinal samples of transporter knockout mice (Supplementary Fig. S2). Slides were counterstained with DAPI (Sigma-Aldrich), 1:1000, and mounted in vectashield (Vector laboratories). Sections were viewed on a Fluoview FV1000 confocal laser microscope (Olympus) or a Leica DMI6000 B inverted fluorescence microscope (Leica Microsystems).

### Permeability

Permeability of fluorescence markers into the lumen of organoids was tested using Na^ + ^-Fluorescein as 4 kDa and Fluorescein isothiocyanate-dextran as 40 kDa tracer (both Sigma-Aldrich). Organoids were washed twice with PBS (pH 7.4) and incubated with the respective fluorescent tracer at final concentration of 1.25 μM for 30 min at room temperature. Marker solutions were removed and organoid cultures were washed min. 5 times with PBS.

### Substrate transport and inhibition studies

For transport studies, organoids generated from wild type or transporter-deficient mice were seeded in a 48-well plate with a density of ca. 40 organoids per well. Organoid cultures were washed three times with HEPES-saline buffer containing (in mM) 138 NaCl, 10 HEPES, 4.5 KCl, 4.2 NaHCO_3_, 2.6 CaCl_2_, 1.2 NaH_2_PO_4_, 1.2 MgCl_2_ (pH 6.5 for peptide and cefadroxil uptake, pH 7.4 for all other transport experiments), and incubated with the respective substrate/inhibitor solutions for 30 min at 37 °C. Transporter activities were assessed using (non-labeled) substrates and ^14^C-radiolabeled substrate tracers (all American Radiolabeled Chemicals). Organoids were preincubated with the respective inhibitor prior to addition of the substrate solutions containing radiolabeled tracers. The following final substrate concentrations were used: 3 mM D-glucose (final concentration of radiolabeled tracer 280 μM, specific activity 10 mCi/mmol), 1 mM α-MDG (radiolabeled tracer 33 μM, specific activity 300 mCi/mmol), 10 mM D-fructose (radiolabeled tracer 10 μM, specific activity 300 mCi/mmol), 5 mM Gly-Sar (radiolabeled tracer 1 mM, specific activity 56 mCi/mmol). Final concentration of competitors and inhibitors were 10 mM Gly-Gly, 20 mM Cefadroxil, 1 mM Phloridzin and 1 mM Phloretin.

Following incubation, substrate/inhibitor solutions were removed and organoid cultures were washed twice with PBS (pH 7.4). Organoids were washed two more times by resuspending cultures (incl. Matrigel) in cold PBS and centrifugation for 10 min at 300 *g.* Organoid pellets were resuspended in a lysis buffer containing 150 mM NaCl, 50 mM Tris-HCl, 1% Igepal CA-630, 0.5% DCA and a protease inhibitor mix, for 20 min at 37 °C. 3 ml of Bioscint® Scintillation solution (National Diagnostics) were added to the cell lysate and radiolabeled substrate taken up by organoid enterocytes was quantified using a liquid scintillation counter (PerkinElmer). Residual amounts of all radiolabeled tracers were measured in control samples (matrigel alone, without organoids) and were found to be negligible (<1%) compared to the substrates accumulated and measured in the epithelial cells of the organoid.

### RNA isolation, reverse transcription and real-time PCR

For RNA isolation, organoids were harvested by dissolving Matrigel including organoids with cold PBS. Following centrifugation at 300 *g* for 5 min at 4 °C, supernatant was removed and pelleted organoids were carefully resuspended in 350 μl of RLT buffer (Qiagen). Total RNA was isolated using the RNeasy Mini Kit (Qiagen) according to the manufacturer’s instructions. RNA concentration and purity (A260/A280 ratio) was determined by spectrophotometric analysis (ND-1000 spectrophotometer, NanoDrop Technologies). Reverse transcription was performed using 200 ng total RNA. Complementary DNA was prepared using Super-Script™ First-Strand Synthesis System (Life Technologies). Quantification of gene expression was performed on Light-Cycler® 480 System using the Universal Probe Library System (Roche Diagnostics) according to the manufacturer’s instructions. Relative induction of gene mRNA expression was calculated using the expression of *Hprt* or *18s* for normalization. PCR products were subjected to electrophoresis on 2% agarose gels to document amplicon specificity. The following primers were used: For *Gcg* forward primer 5′-tggttggaaccttggtgaata-3′ and reverse primer 5′-gctgcagcccattaagatg-3′ (Probe#58), for *Gip* forward primer 5′-tctcagggaaaggaggacaa-3′ and reverse primer 5′-tcagcacatcatcatcactgag-3′ (Probe#98), for *Hprt* forward primer 5′-tcctcctcagaccgctttt-3′ and reverse primer 5′-cctggttcatcatcgctaatc-3′ (Probe#95), and for *18 s* forward primer 5′-aaatcagttatggttcctttggtc-3′ and reverse primer 5′-gctctagaattaccacagttatccaa-3′ (Probe#55).

### Incretin hormone secretion

Organoids generated from wild type or transporter-deficient mice were seeded in a 48-well plate (density ca. 40 organoids per well as for uptake experiments). Organoid cultures were washed four times with the same HEPES-saline buffer (pH 7.4) as used for transport studies, additionally containing BSA and DPP4 inhibitor. Cultures were incubated 2 hours at 37 °C with either HEPES-saline buffer (for basal hormone secretion), 50 mM D-glucose, 50 mM Gly-Sar, 30 μM deoxycholic acid or a mixture of 10 μM Forskolin and 10 μM 3-isobutyl-1-methylxanthine (for maximal hormone output). The following experimental procedures were performed on ice. The culture plate was placed on ice, supernatants were collected and immediately, cold PBS was added to the organoid cultures (see transport studies). Secretion samples were centrifuged (400 *g*) for 5 min at 4 °C and the supernatant was snap-frozen in liquid nitrogen. Organoids including Matrigel were resuspended in cold PBS and washed twice by centrifugation (600 *g*) for 10 min at 4 °C. The supernatant was collected and snap-frozen. All samples were kept at −80 °C until analysis of incretin hormones. GLP-1 and GIP concentrations in secretion and lysate samples were quantified using a Glucagon-Like Peptide 1 (Active) ELISA kit and a Rat/Mouse GIP (Total) ELISA kit (both Millipore).

The amount of GLP-1 secreted by the cultures was calculated as a percentage of the GLP-1 content (secreted plus intracellular) and normalized to basal secretion. Basal GLP-1 secretion of organoid cultures accounts for ca. 1% and basal GIP secretion for ca. 1.5% (amount secreted per total hormone content of the cultures). Total hormone content (secreted in supernatant plus intracellular in cell lysate) of the organoid cultures was ca. 250 fmol GLP-1 and 20 pmol GIP per well (ca. 40 organoids).

### Calcium imaging using Fura-2-AM

Calcium-imaging experiments were conducted in a 48-well plate. Organoid cultures were loaded with 7 μM Fura-2 AM (Life Technologies) in HEPES-saline buffer (see transport studies) containing 0.01% pluronic F127, 375 μM eserine and 2 mM probenecid and incubated for 15 min at 37 °C followed by 15 min incubation at room temperature (RT). Organoid cultures were washed 5 times with PBS (pH 7.4; 37 °C) and images were recorded using a Leica DMI6000 B inverted fluorescence microscope (Leica Microsystems) at 20× magnification every 5–10 s. The fluorescence ratio F(λ_ex_340 nm)/F(λ_ex_380 nm) was calculated and imaging data were analyzed using the Leica Application Suite Advanced Fluorescence software. All imaging experiments were performed minimum 6 times and representative time courses are depicted in the manuscript.

### Imaging of intracellular acidification using BCECF-AM

For imaging of intracellular acidification, organoids were seeded in a 48-well plate. Organoid cultures were incubated with 1 μM BCECF AM (Life Technologies) in HEPES-saline buffer as used for calcium imaging for 20 min at 37 °C and then washed 5 times with PBS (pH 7.4; 37 °C). Image series were captured every 3–7 s and the fluorescence ratio F(λ_ex_490 nm)/F(λ_ex_450 nm) was calculated. Calculations and imaging data analyses were performed using the Leica Application Suite Advanced Fluorescence software.

### Statistical analysis

All statistical computations were performed using SigmaStat software from Systat Software that tests for normal distribution and equal variance. Data comparing two groups were analyzed using unpaired t test. Data comparing several treatments/genotypes vs corresponding control group were analyzed using One-Way or Two-Way analysis of variance (ANOVA) followed by an appropriate multiple comparison procedure. Supplementary Table S1 comprises statistical tests and P-values for all data/groups compared. Data are expressed as mean ± SEM, n ≥ 5 for all groups unless otherwise stated. Differences between groups were considered significant if P-values were < 0.05. (*) indicates P-values < 0.05, (**) indicates P-values < 0.01, (***) indicates P-values < 0.001.

## Additional Information

**How to cite this article**: Zietek, T. *et al.* Intestinal organoids for assessing nutrient transport, sensing and incretin secretion. *Sci. Rep.* **5**, 16831; doi: 10.1038/srep16831 (2015).

## Supplementary Material

Supplementary Information

Supplementary video S1

Supplementary video S2

Supplementary video S3
