# Supplementary material for: Intestinal organoids for assessing nutrient transport, sensing and incretin secretion
Source: Sci Rep. 2015 Nov 19;5:16831. doi: 10.1038/srep16831 (PMC4652176; doi:10.1038/srep16831)
Supplement: Supplementary Information [file srep16831-s1.pdf]

Supplementary information

Manuscript title:
Intestinal organoids for assessing nutrient transport, sensing and incretin secretion

Authors list:
Tamara Zietek, Eva Rath, Dirk Haller, Hannelore Daniel

Supplementary Table S1

Supplementary Table S1: Statistical analyses of transport and hormone secretion studies. Overview of statistical analyses and calculated p-values for the data depicted in **Fig. 1c-e**, **Fig. 2b-d** and **Fig. 2f,g**.

Figure	Comparison	Test	P-Value
**1c left**	WT Glucose vs. WT Glucose + Phloridzin	Two Way ANOVA followed by Tukey test	<0.001
	WT Glucose vs. SGLT1$^{-/-}$ Glucose		<0.001
	SGLT1$^{-/-}$ Glucose vs. SGLT1$^{-/-}$ Glucose + Phloretin		0.040
**1c mid**	WT α-MDG vs. WT α-MDG + Phloridzin	Two Way ANOVA followed by Tukey test	<0.001
	WT α-MDG vs. SGLT1$^{-/-}$ α-MDG		<0.001
**1c right**	WT Fructose vs. GLUT5$^{-/-}$ Fructose	Two Way ANOVA followed by Tukey test	<0.001
	GLUT5$^{-/-}$ Fructose vs. GLUT5$^{-/-}$ Fructose + Phloretin		0.018
**1d**	WT Gly-Sar vs. PEPT1$^{-/-}$ Gly-Sar	Student´s t test (unpaired)	0.008
**1e**	WT Gly-Sar vs. WT Gly-Sar + Gly-Gly	One Way ANOVA followed by Holm-Sidak method	0.009
	WT Gly-Sar vs. WT Gly-Sar + Cefadroxil		<0.001
**2b**	Duodenum vs. Jejunum	One Way ANOVA followed by Holm-Sidak method	0.034
	Duodenum vs. Ileum		<0.001
	Jejunum vs. Ileum		<0.001
**2c**	Basal vs. Glucose	One Way ANOVA followed by Holm-Sidak method	<0.001
	Basal vs. Gly-Sar		0.013
	Basal vs. DCA		<0.001
**2d left**	WT Basal vs. Gly-Sar	One Way ANOVA followed by Holm-Sidak method	0.006
	WT Basal vs. F/I		<0.001
	PEPT1$^{-/-}$ Basal vs. Gly-Sar	One Way ANOVA followed by Holm-Sidak method	**0.876**
	PEPT1$^{-/-}$ Basal vs. F/I		<0.001
	WT F/I vs. PEPT1$^{-/-}$ F/I	Two Way ANOVA followed by Tukey test	**0.961**
**2d right**	WT Basal vs. Glucose	One Way ANOVA followed by Holm-Sidak method	0.003
	WT Basal vs. F/I		<0.001
	SGLT1$^{-/-}$ Basal vs. Glucose	One Way ANOVA followed by Holm-Sidak method	**0.964**
	SGLT1$^{-/-}$ Basal vs. F/I		<0.001
	WT F/I vs. SGLT1$^{-/-}$ F/I	Two Way ANOVA followed by Tukey test	**0.743**
**2f**	Duodenum vs. Jejunum	One Way ANOVA followed by Holm-Sidak method	<0.001
	Duodenum vs. Ileum		<0.001
	Jejunum vs. Ileum		<0.001
**2g**	WT Basal vs. Glucose	One Way ANOVA followed by Holm-Sidak method	0.043
	WT Basal vs. F/I		<0.001
	SGLT1$^{-/-}$ Basal vs. Glucose	One Way ANOVA followed by Holm-Sidak method	**0.746**
	SGLT1$^{-/-}$ Basal vs. F/I		<0.001
	WT F/I vs. SGLT1$^{-/-}$ F/I	Two Way ANOVA followed by Tukey test	**0.438**

Supplementary Figure S1

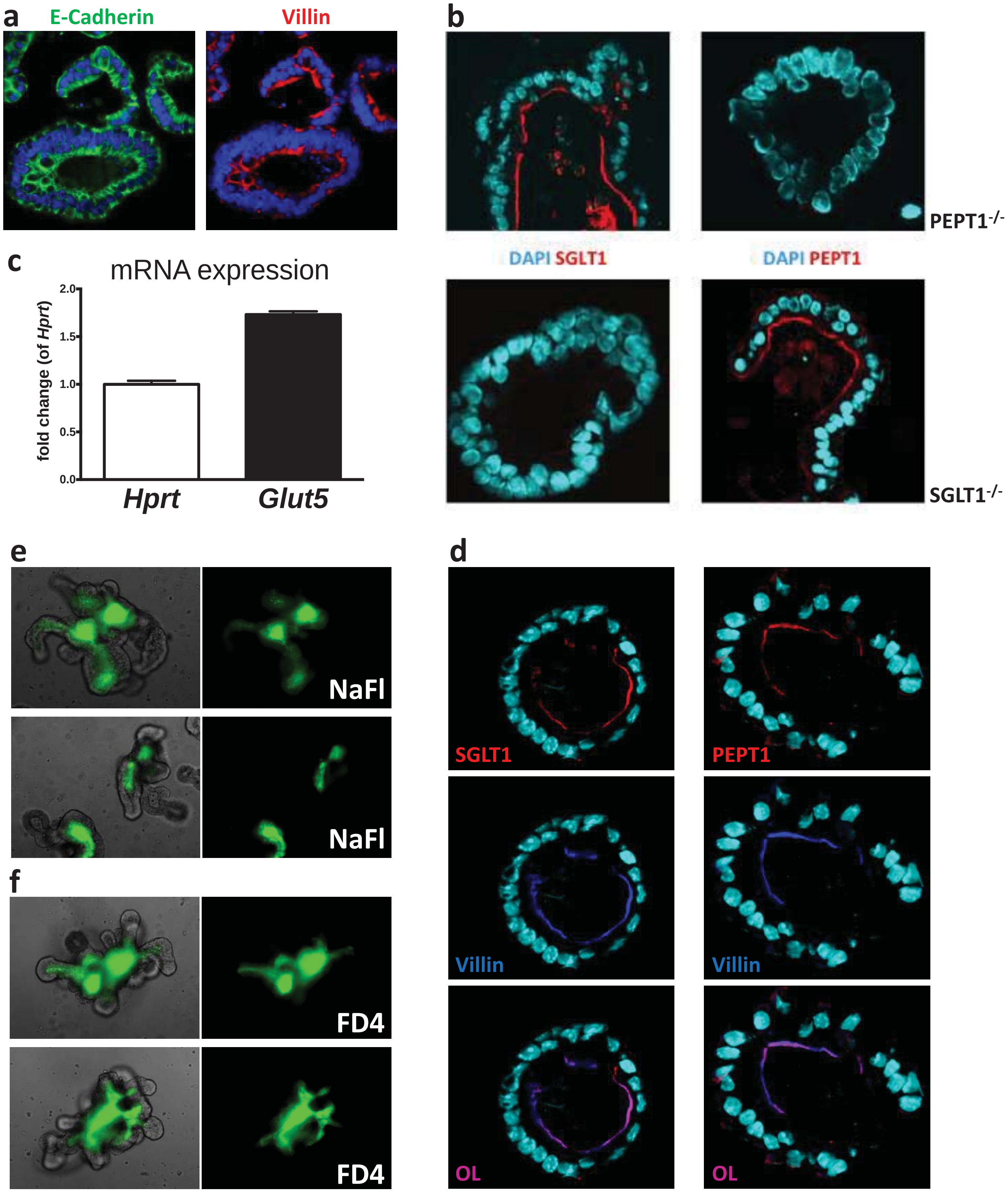

Supplementary Figure S1: Cell polarization, specificity of PEPT1 and SGLT1 antibodies, *Glut5* expression, and permeability of the organoid epithelium.

(a) Immunofluorescent staining of villin (red) and E-Cadherin (green) shows polarized intestinal epithelial cells; nuclei (blue). **(b)** Organoids derived from PEPT1$^{-/-}$ (upper panel) and SGLT1$^{-/-}$ (lower panel) mice demonstrate no staining to the corresponding PEPT1 or SGLT1 antibodies, respectively. As a control, PEPT1$^{-/-}$ organoids show specific SGLT1 staining and vice versa. **(c)** *Glut5* mRNA expression in SI organoids derived from WT mice. mRNA levels of the housekeeping gene *Hprt* serve as control. **(d)** Apical colocalization of villin and transporters. **(e, f)** Permeability of sodium fluorescein (NaFl) and FITC-Dextran 4 kDa (FD4), both accumulating in the organoid lumen.

Supplementary Figure S2

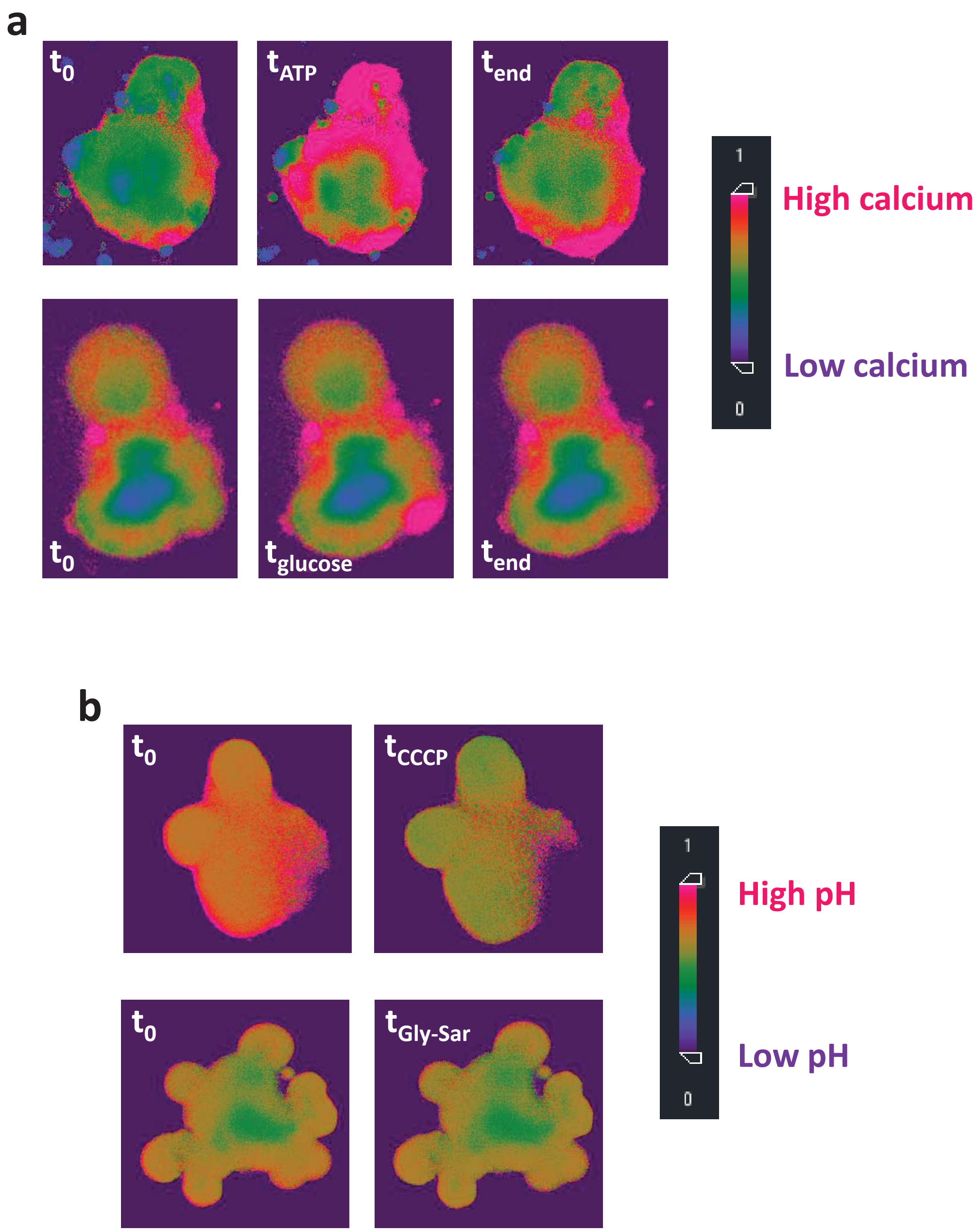

Supplementary Figure S2: Live-cell imaging of intracellular calcium and acidification in WT organoids. (a) Calcium responses to 100 µM ATP (upper panel) and 50 mM glucose (lower panel) in WT organoids. Ratio of fluorescence intensities F (λ_{ex}340 nm)/F (λ_{ex}380 nm) display changes in intracellular calcium levels according to the color legend. **(b)** Intracellular acidification of organoid epithelial cells upon treatment with the protonophore CCCP (upper panel) and 50 mM Gly-Sar (lower panel), ratio images. Ratio of fluorescence intensities F (λ_{ex}490 nm)/F (λ_{ex}450 nm) display changes in intracellular calcium levels according to the color legend.
